# VEGF Mediates Retinal Müller Cell Viability and Neuroprotection through BDNF in Diabetes

**DOI:** 10.3390/biom11050712

**Published:** 2021-05-10

**Authors:** Yun-Zheng Le, Bei Xu, Ana J. Chucair-Elliott, Huiru Zhang, Meili Zhu

**Affiliations:** 1 Section of Endocrinology, Diabetes and Metabolism, Department of Medicine, University of Oklahoma Health Sciences Center, Oklahoma City, OK 73104, USA; xu_bei1017@163.com (B.X.); ana-chucair@omrf.org (A.J.C.-E.); zhr67@163.com (H.Z.); Meili-Zhu@ouhsc.edu (M.Z.); 2 Department of Cell Biology, University of Oklahoma Health Sciences Center, Oklahoma City, OK 73104, USA; 3 Department of Ophthalmology, University of Oklahoma Health Sciences Center, Oklahoma City, OK 73104, USA; 4 Harold Hamm Diabetes Center, University of Oklahoma Health Sciences Center, Oklahoma City, OK 73104, USA; 5 Eye Center of Xiangya Hospital, Central South University and Hunan Key Laboratory of Ophthalmology, Changsha 410008, China; 6 College of Biological Engineering, Henan University of Technology, Zhengzhou 450001, China

**Keywords:** Müller glia, diabetic retinopathy, neuroprotection, VEGF, BDNF, TRK-B, AKT, ERK

## Abstract

To investigate the mechanism of vascular endothelial growth factor (VEGF) and brain-derived neurotrophic factor (BDNF) in Müller cell (MC) viability and neuroprotection in diabetic retinopathy (DR), we examined the role of VEGF in MC viability and BDNF production, and the effect of BDNF on MC viability under diabetic conditions. Mouse primary MCs and cells of a rat MC line, rMC1, were used in investigating MC viability and BDNF production under diabetic conditions. VEGF-stimulated BDNF production was confirmed in mice. The mechanism of BDNF-mediated MC viability was examined using siRNA knockdown. Under diabetic conditions, recombinant VEGF (rVEGF) stimulated MC viability and BDNF production in a dose-dependent manner. rBDNF also supported MC viability in a dose-dependent manner. Targeting BDNF receptor tropomyosin receptor kinase B (TRK-B) with siRNA knockdown substantially downregulated the activated (phosphorylated) form of serine/threonine-specific protein kinase (AKT) and extracellular signal-regulated kinase (ERK), classical survival and proliferation mediators. Finally, the loss of MC viability in *TrkB* siRNA transfected cells under diabetic conditions was rescued by rBDNF. Our results provide direct evidence that VEGF is a positive regulator for BDNF production in diabetes for the first time. This information is essential for developing BDNF-mediated neuroprotection in DR and hypoxic retinal diseases, and for improving anti-VEGF treatment for these blood–retina barrier disorders, in which VEGF is a major therapeutic target for vascular abnormalities.

## 1. Introduction

Müller cells (MCs) are located in close proximity to almost all retinal functional components and cellular entities, including photoreceptors, secondary neurons, ganglion cells, vasculature, and vitreous. This anatomical arrangement is ideal for MCs to serve as major retinal supporting cells that perform many essential functions, such as ion and water transport, energy metabolism, visual pigment recycling, synaptic activity, neural transmission, structural support and insulation, pathogen removal, anti-oxidative effect, blood–retina barrier (BRB) maintenance, cytokine and trophic factor production, neuroprotection, and neural progenitors and retinal regeneration (for review, see [1,2,3,4]). With these functionalities, MCs serve well as a major checkpoint for retinal health and homeostasis under physiological conditions. In diabetes, MCs respond to hyperglycemia/high retinal glucose and upregulate the production of inflammatory cytokines, BRB permeable factors, and neurotrophic factors, either by themselves or in conjunction with other cellular entities. It has been suggested that the accumulation of advanced lipoxidation end-products and glycation end-products in MCs is a contributing factor for MC dysfunction during the pathogenesis of diabetic retinopathy (DR) [5,6,7], a neurovascular disorder and a leading cause of blindness (for review, see [8]). In the development of DR, MCs play a causative role in diabetes-induced upregulation of tumor necrosis factor-α and vascular endothelial growth factor-A (VEGF-A or VEGF) [9,10,11], which leads to retinal hypoxia, oxidative stress, BRB lesions and breakdown, and retinal neurovascularization, major pathological characteristics of DR. Finally, MCs are a major participant in regulating water balance through the potassium channels [12], which is potentially relevant to diabetic macular edema, a major vision loss in DR. For the past three decades, intensive clinical, pathobiological, and pharmacological studies have identified VEGF as a major therapeutic target for BRB breakdown in DR, age-related macular degeneration (AMD), and other hypoxic retinal vascular diseases (for review see [13]), which has led to the development of anti-VEGF drugs as a major therapeutic strategy for these disorders. While anti-VEGF drugs are effective in reducing BRB pathology and improving visual acuity in some patients [14,15], the average gain of visual acuities is not sustained for patients with long-term anti-VEGF treatment for neovascular AMD and diabetic macular edema [16,17,18]. Retinal, choroidal, and scleral thinning, and retinal pigment epithelium tear have also been reported among patients with long-term anti-VEGF treatments [17,18,19,20,21,22,23]. While long-term (five years or longer) clinical trial data on anti-VEGF drugs for DR are not available, the observations that a VEGF neutralizing antibody and shRNA cause retinal degeneration in experimental diabetes and retinopathy of prematurity [24,25] suggest a possibility of having adverse effects in some long-term anti-VEGF drug-treated patients.

To address whether VEGF signaling in MCs is critical to retinal MC and neuronal integrity, as proposed in the field [26], we generated MC-specific VEGF receptor-2 (VEGFR2) knockout mice using MC Cre-driver line generated in our laboratory [27,28]. We revealed that the VEGFR2-mediated AKT survival pathway was a major contributor to MC viability in diabetes, which, in turn, provides neuroprotection in diabetic animals [27]. Interestingly, the streptozotocin-induced diabetic MC-specific VEGFR2 knockout mice had accelerated loss of brain-derived neurotrophic factor (BDNF) [27]. BDNF belongs to a family of neurotrophins that have been shown to act as trophic factors for both the central and peripheral nerve systems. MCs have been suggested as a major cellular source of retinal BDNF and other neurotrophins [29,30]. Although the role of neurotrophins in protecting ganglion cells under various stresses has been explored [31,32,33], little is known about the function of this family of trophic factors in diabetic and hypoxic retinas. It has been suggested that neurotrophins may play a role in keeping vascular integrity [4], which is in agreement with the suggestion that BDNF is a potential biomarker for DR [34,35]. Our observation of an accelerated reduction in the retinal BDNF level from diabetic MC-specific VEGFR2 knockout mice clearly suggests that, except its own direct role in neuroprotection, VEGF signaling provides additional trophic effect for MCs and all types of neurons through the production and action of MC-derived neurotrophins, including BDNF. Therefore, there is a potential therapeutic benefit for targeting BDNF in MCs for neuroprotection, which will not only be important generally for treating neuronal degeneration in DR, AMD, and other hypoxic retinal diseases, but will also be particularly useful in dealing with the potential retinal degeneration reported in patients with long-term anti-VEGF therapies [16,19]. While a conditional gene knockout approach is capable of revealing information on MC-directed physiological consequences, dissecting their underlying mechanisms is challenging, as MCs comprise a small percentage of retinal cells. We thus established cellular systems for rapid testing of the efficacy of neuroprotective agents for MC viability, investigating biochemical mechanism for promoting MC viability, formulating a working hypothesis, and developing MC-mediated neuroprotective strategies [36]. This article is a summary of our work on investigating the relationship between VEGF- and BDNF-mediated MC viability in diabetes, the role and mechanism of BDNF in this process, and the therapeutic potential of BDNF-mediated MC survival and neuroprotection in DR and hypoxic BRB disorders.

## 2. Materials and Methods

### 2.1. Animal Treatment and Use

Animal work was performed according to the guidelines in the statement for the “Use of Animals in Ophthalmic and Vision Research” established by the Association for Research in Vision and Ophthalmology”. The animal protocol (19-057-EFCHI) was approved by the Institutional Animal Care and Use Committee of the University of Oklahoma Health Sciences Center. Intravitreal injection was performed using 1-month old C57Bl6 background mice (4–6 animals for each group). Immediately after the mice were anesthetized, one drop of 5% tropicamide and a drop of 0.5% proparacaine were applied to the eye for dilation and local anesthesia, respectively. rVEGF (0.2 µg in 1 µL) or vehicle control was intravitreally injected at a location 1 mm posterior to the limbus using a syringe with a 32-gauge needle, according to previous procedures [37,38]. One day after intravitreal injection, the animals were euthanized and their eyes were collected, frozen with liquid nitrogen, and stored at −70 °C before analysis.

### 2.2. Cell Cultures, Viability Assay, and Transfection

Rat Müller cell line rMC1 was provided by Dr. V. P. Sarthy [39] and was routinely subjected to authentication by immunocytochemical analysis with antibodies against glutamine synthetase (GS), an MC-specific marker. Mouse primary MCs were prepared according to previous procedures [9,11]. Primary MCs (up to passage 6) were used in the experiments. Primary MCs and rMC1 cells were maintained in DMEM medium with 1 g/L glucose, supplemented with 10% fetal bovine serum (FBS) and 1% penicillin–streptomycin. For experimental tests, cells were seeded with an appropriate concentration and incubated overnight, followed by serum starvation for additional 4 to 6 h before experiments. The cells were than exposed to high glucose (HG, 25 mmol/L) or normal glucose (NG, 5 mmol/L with mannitol (25 mmol/L)) containing 0.25% FBS for various durations, depending on experimental design. Cells were also grown on glass slides or Falcon culture slides (#62405-178) from VWR (Radnor, PA, USA) for immunocytochemical staining. Cell viability assay was performed in 96-well plates using a cell cytotoxicity assay kit (ab112118) from Abcam (Cambridge, MA, USA), based on the manufacturer’s instruction. Briefly, 1.5 × 10^4^ cells were seeded in each well and were subjected to various treatments in 100 µL media. After the treatment, 20 µL of proprietary dye solution was added and incubated at 37 °C for 1 h; the ratio of OD_570_ vs. OD_605_ is used to represent cell viability. The cells were subjected to HG or NG treatment, in the presence or absence of rVEGF (hBA-165, #SC-4571) or BDNF (hBA-238, #SC-4554 BDNF), which were purchased from Santa Cruz Biotechnology (Dallas, TX, USA). rVEGF (#293-VE) and rBDNF (#248-BD) from R&D Systems (Minneapolis, MN, USA) were used for confirming bioactivities. siRNA transfection in rMC1 cells was carried out using siRNA Reagent System (#SC-45064) and rat *TrkB* siRNA (#SC-270466) from Santa Cruz Biotechnology (Dallas, TX, USA), according to the manufacturer’s instruction. Live cells were also confirmed with 4,6-diamidino-2-phenylindole (DAPI) from Thermo Fisher (Waltham, MA, USA).

### 2.3. Gene Expression Analysis, Immunoblotting, Immunocytochemistry, and ELISA

For quantitative RT-PCR, total RNA was prepared with Qiagen RNeasy Mini Kit (Qiagen, Germantown, MD, USA), according to the manufacturer’s instruction. The first strand cDNA was synthesized by random hexamer priming with the SuperScript^TM^ first strand synthesis system for RT-PCR (Thermo Fisher, Waltham, MA, USA), after a brief treatment of RNA samples with amplification grade DNase I (Thermo Fisher, Waltham, MA, USA). The qRT-PCR was carried out with a CFX96 qRT-PCR instrument (BioRad, Hercules, CA, USA) using cDNA, iQ SYBR Green Supermix (BioRad, Hercules, CA, USA), and sequence-specific primers, with 40 cycles of denaturing (94 °C for 15 s), annealing (at a temperature determined by the specific primer pair, for 30 s), and extension (72 °C for 30 s), followed by melt-curve analysis. For *TrkB* mRNA quantification, primer pair 5-CGG AGT GCT ATA ACC TCT GC-3′ and 5′-GAA CTT GAC AAT GTG CTC GTG-3′ (annealing temperature: 60 °C) was used, according to rat cDNA sequence from gene bank (accession: NM_012643). The level of *TrkB* mRNA was normalized to β-*Actin* mRNA using the following primer pair: 5′-GCC GGC TTC GCG GGC GAC GA-3′ and 5′-GCC ACA CGC AGC TCA TTG TAG A-3′ (annealing temperature: 60 °C) for quantification. Results were analyzed with the comparative Ct method, according to the manufacturer’s instruction.

Protein expression was measured by immunoblotting and immunocytochemistry as described previously [9,27] with primary antibodies against β-actin (#A5441), GS (#MAB302) from Sigma (St. Louis, MO, USA), GS (#Pa1-46165), and BDNF (#OSB00017 W) from Thermo Fisher (Waltham, MA, USA), BDNF (#SC-546) and proBDNF (#SC-65514) from Santa Cruz Biotechnology (Dallas, TX, USA), AKT (#4064), pAKT (#9271), ERK (#9101), and pERK (#9102) from Cell Signaling Technology (Beverly, MA, USA), and TRK-B (#ab18987) from Abcam (Cambridge, MA, USA). The dilution rate for primary antibodies was based on manufacturer’s instructions, with or without modification after an initial test (Table 1). The horseradish peroxidase-conjugated secondary antibodies for immunoblotting were from Vector Lab (Burlingame, CA, USA). For immunocytochemistry, all fluorescent secondary antibodies were obtained from Thermo Fisher (Waltham, MA, USA). For immunocytochemical analysis of bromodeoxyuridine (BrdU), the antibody (#B2531) was purchased from Sigma (St. Louis, MO, USA). Cells were immersed in BrdU (10 µM) containing media for at least 2.5 h before the initiation of analysis. The dilution factors for primary and secondary antibodies in immunoblotting and immunohistochemistry are listed in Table 1. Measurement of BDNF concentration with enzyme-linked immunosorbent assay (ELISA) was performed using a Due ELISA Set (#DY248) from R&D System (Minneapolis, MN, USA), according to the manufacturer’s instruction.

### 2.4. Statistical Analyses

Data were expressed as mean ± SD (or SEM). Statistical analysis was performed with pairwise *t*-test or one-way ANOVA, with *p* < 0.05 considered statistically significant. For data requiring statistical analysis, at least three data points were used, and the experiment was repeated at least once.

## 3. Results

### 3.1. VEGF-Stimulated MC Viability

**Figure 1:**
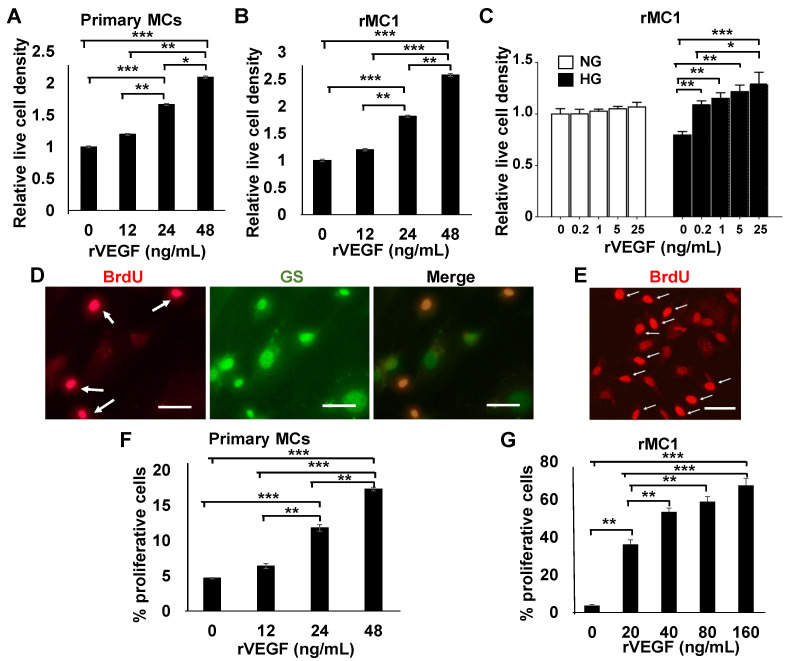
rVEGF-stimulated MC viability in mouse primary cells and rMC1 cells cultured in high HG media. (**A**) Relative live cell density of primary MCs cultured for 72 h on slides in HG media with and without rVEGF. (**B**) Relative live cell density of rMC1 cells cultured on slides for 36 in HG media with or without rVEGF. (**C**) Live cell analysis for rMC1 cells cultured for 36 h in NG or HG media with or without rVEGF supplement. (**D**,**F**) Images and quantifications of BrdU (red) stained (white arrows) primary MCs cultured for 24 h, in HG media with and without rVEGF supplement (24 ng/mL). (**E**,**G**) Images and quantifications of BrdU stained (white arrows) rMC1 cells cultured for 3 h, in HG media with and without rVEGF supplement (20 ng/mL). GS: Glutamine synthetase, Müller glial marker. Scale bars: 50 µM. Graph bars represent mean ± SEM. *: *p* < 0.05; **: *p* < 0.01; ***: *p* < 0.001. rVEGF was capable of stimulating primary MC survival and proliferation in a dose-dependent manner under diabetes-like condition.

To determine the effect of VEGF on MC viability, mouse primary MCs were cultured in HG media supplemented with rVEGF, and the number of live cells (DAPI-stained) was scored. Figure 1A shows that rVEGF was capable of stimulating primary MC viability in a dose-dependent manner. This viability was contributed to by VEGF-stimulated primary MC proliferation in a dose-dependent manner (Figure 1D,F). As the quantities of primary MCs are limited, we aimed to recapitulate these observations using a rat cell line rMC1 [39], with a goal of performing more analysis from significantly more cellular material. rMC1 cells are derived from rat primary MCs and express common MC markers, such as glial fibrillary acidic protein, cellular retinaldehyde-binding protein, vimentin, and GS [39,40,41]. This is very similar to that in primary murine MCs [11,27,42] and other murine and human MC lines, such as MIO-M1, QMMuC-1, and SIRMu-1 MC lines [42,43,44]. Therefore, the results obtained in rMC1 cells should carry general Müller glial characteristics demonstrated by most murine primary MCs, and murine and human MC lines. As a result, rMC1 cells have been successfully used in more than 100 MC pathobiological studies, according to PubMed. We have utilized rMC1 cells for various work in our laboratory for the past decade and have accumulated a significant amount of hands-on experience about its usage and reagent requirements [36,45]. In our hands, rVEGF was capable of rescuing the loss of rMC1 cells cultured in HG media in a dose-dependent manner (Figure 1B,C). Likewise, these cells also demonstrated the rVEGF-mediating proliferation in a dose-dependent manner (Figure 1E,G).

### 3.2. VEGF-Stimulated BDNF Synthesis and Production

**Figure 2:**
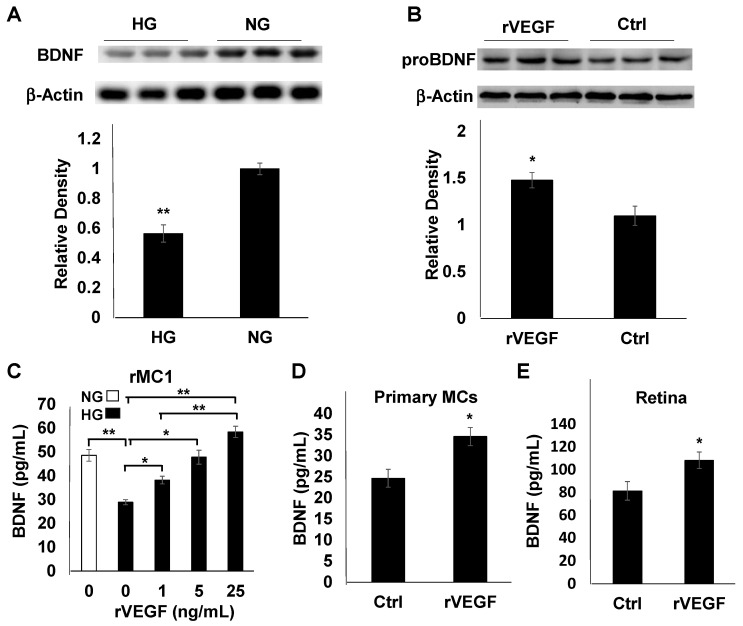
BDNF production in rMC1 cells and mouse primary MCs and retinas. (**A**) Immunoblotting analysis of BDNF synthesis using cell-free extracts from rMC1 cells cultured in NG and HG media for 48 h. (**B**) Immunoblotting analysis of rVEGF (20 ng/mL)-stimulated proBDNF synthesis using cell-free extracts from rMC1 cells cultured in NG media for 48 h. (**C**) ELISA analysis of VEGF-stimulated BDNF accumulation in culture supernatants of rMC1 cells grown in HG media for 48 h. (**D**) ELISA analysis of VEGF-stimulated BDNF accumulation in culture supernatants of primary MCs cells grown in HG media for 48 h. (**E**): ELISA analysis of retinal BDNF in 1 month old C57B6 background mice 1 day after intravitreal injection of rVEGF (0.2 µg/eye). Graph bars represent mean ± SEM. *: *p*: 0.05; **: *p* < 0.01. VEGF upregulated BDNF production in MCs and mouse retinas.

We showed previously that diabetes downregulated retinal BDNF accumulation, and disruption of VEGFR2 in Müller glia caused an accelerated loss of retinal BDNF in diabetic mice [27]. To determine if MCs are the cellular target for the regulation of BDNF production in diabetes, we examined the synthesis of BDNF in rMC1 cells. Immunoblotting analysis of cell extracts indicated that BDNF accumulation was reduced in rMC1 cells cultured in HG media (Figure 2A). We then investigated if VEGF was capable of upregulating BDNF synthesis in MCs. As excessive BDNF inside the MCs would most likely be secreted, we examined the levels of proBDNF in rMC1 cell extracts. rVEGF supplement resulted in an increase in proBDNF accumulation inside rMC1 cells cultured in NG media (Figure 2B). ELISA analysis of culture supernatants from rMC1 cells indicated that, while diabetes-like conditions (HG) significantly downregulated BDNF production, rVEGF stimulated the BDNF secretion in a dose-dependent manner (NG, Figure 2C). This observation was confirmed by a similar experiment in primary MCs cultured in HG media (Figure 2D). For an in vivo confirmation, we performed ELISA analysis of retinal extracts from mice 24 h after being injected intravitreally with rVEGF (200 ng). The rVEGF-injected retinas demonstrated an elevated level of BDNF production (Figure 2E).

### 3.3. BDNF-Stimulated MC Viability and Potential Mechanism

**Figure 3:**
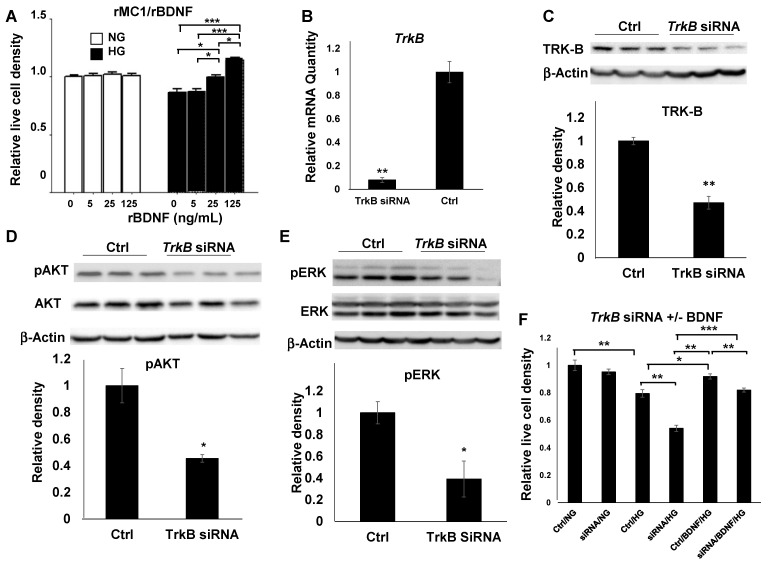
BDNF-mediated MC viability. (**A**) Viability of rMC1 cells cultured in NG or HG media with or without rBDNF. **A**: rBDNF-mediated rMC1 cell viability in a dose-dependent manner under a diabetes-like condition. (**B**,**C**) Quantitative RT-PCR and immunoblotting analysis of rMC1 cells 48 h after *TrkB* siRNA and scramble siRNA control (Ctrl) transfection. (**D**,**E**) Analysis and representative images of immunoblotting for pAKT and tAKT (**D**) and pERK and tERK (**E**) in rMC1 cells transfected with *TrkB* siRNA and scramble control siRNA 48 h post-transfection in HG media. (**F**) rBDNF (25 ng/mL) rescued HG-induced loss of rMC1 cells transfected with *TrkB* siRNA and scramble control siRNA. Graph bars represent mean ± SEM. *: *p* < 0.05; **: *p* < 0.01; ***: *p* < 0.001. *TrkB* knockdown significantly reduced its mRNA and protein levels and MC viability under diabetic conditions.

To address if BDNF played a direct role in MC viability, we examined the effect of rBDNF on rMC1 cells. Live cell analysis demonstrated that, while diabetes-like conditions (HG) resulted in a loss of rMC1 cells, rBDNF significantly increased the MC viability in a dose-dependent manner (Figure 3A). Although BDNF is a major trophic factor, its underlying mechanism for MC viability and neuroprotection in DR is not well studied. To examine the potential mechanism of BDNF-mediated MC viability, we elected to target the canonical BDNF receptor TRK-B by a genetic approach with siRNA knockdown in rMC1 cells. This siRNA target was selected according to our earlier work [45]. Although the reduction of mRNA was observed from 24 to 96 h post-transfection (data not shown), the maximal levels of reduction were observed 48 h post-transfection. qRT-PCR analysis showed that a reduction of more than 90 percent of mRNA was achieved with *TrkB* siRNA transfection (Figure 3B). This level of mRNA reduction, resulted in a near 50 percent decrease in TRK-B proteins, as determined by immunoblotting (Figure 3C). Unlike the all-or-none expression pattern that occurred in genetic null mutations, siRNAs reduce the level of protein expression in all cells. Therefore, a high degree of mRNA reduction was critical to the successful execution of our goals within the experimental time frames. To determine the effect of *TrkB* knockdown on MC viability and its mechanism, we focused on their putative downstream targets, AKT and ERK, classical mediators for cell survival and proliferation. Transfecting *TrkB* siRNA resulted in a near 50 percent decrease in the level of phosphorylated AKT (pAKT), the activated form of AKT, in rMC1 cells grown in HG media after 48 h, without significant alteration of total AKT protein level (Figure 3D). *TrkB* siRNA transfection also caused a similar level of reduction in phosphorylated ERK (pERK) in rMC1 cells after culture in HG media for 48 h, with no apparent alteration of total ERK level (Figure 3E). These results suggest that a major function of BDNF-mediated signaling through its receptor TRK-B is to support MC survival and proliferation, as demonstrated in the change of pAKT and pERK levels. To test the therapeutic potential of BDNF in protecting MCs in DR, and to rescue the loss of cellular viability by TRK-B in MCs, rBDNF was supplemented to *TrkB* or scramble siRNA transfected rMC1 cells, as performed previously [36]. While TRK-B knockdown caused an insignificant alteration in rMC1 cell viability under normal culture conditions ((NG), Figure 3F), there was a substantial reduction of rMC1 cell viability under diabetic conditions ((HG), Figure 3F). As expected, The *TrkB* siRNA transfected rMC1 cells demonstrated a significant decrease in viability under diabetic conditions (HG), which was reversed partially by rBDNF supplementation. This result suggests that BDNF/TRK-B signaling is a major contributor to MC viability in DR, with potential compensatory mechanism(s).

## 4. Discussions

**Figure 4:**
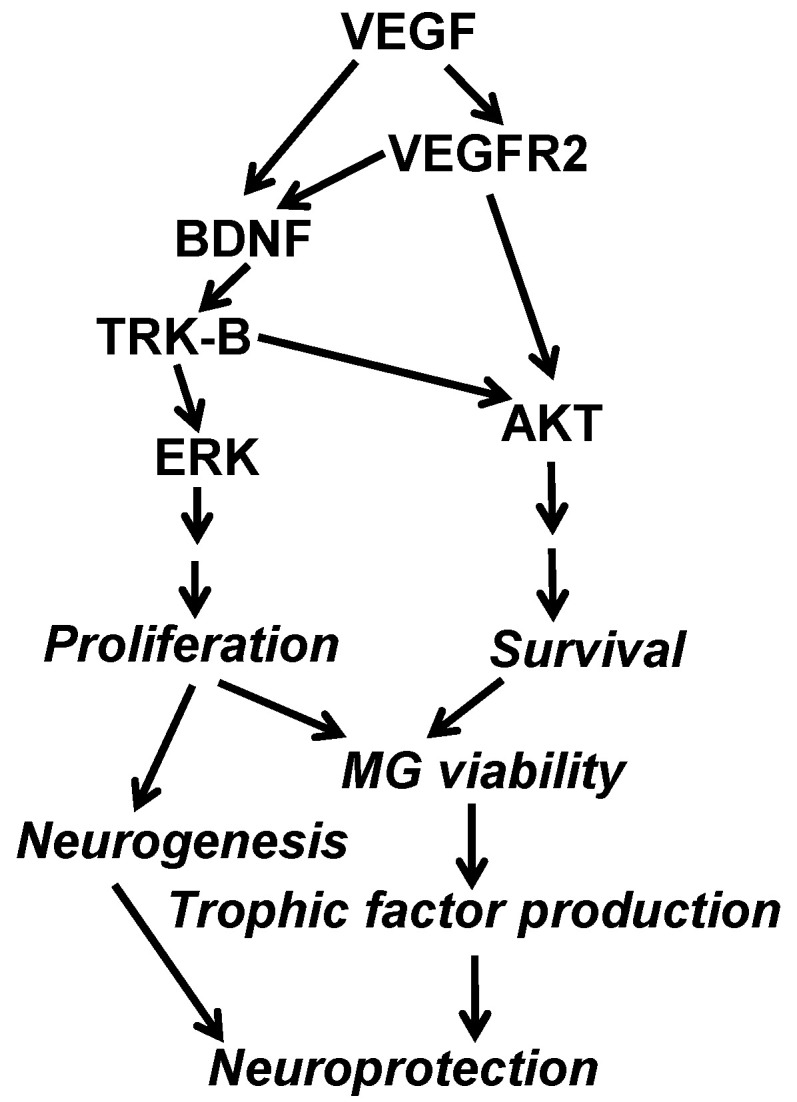
Working hypothesis and mechanisms of VEGF-mediated MC viability and neuroprotection, based on our previous and current work. VEGF plays a cardinal role for neuroprotection in DR and hypoxic retinal disorders through its own action (VEGFR2/AKT-mediated MC survival) and through its downstream neurotrophin-mediated MC viability, including BDNF/TRK-B-mediated MC proliferation and survival. As a result, MCs exert the neuroprotective function by trophic factor production and neurogenesis.

Our initial interests in diabetes/hypoxia-induced neural degeneration and protection was focused on the mechanism of VEGF signaling-mediated protection of MCs in DR and hypoxic retinal diseases. Our work with diabetic MC-specific VEGFR2 knockout mice clearly indicates the cardinal role of the VEGFR2-mediated survival pathway in MC viability and protection [27]. Our finding that there was an accelerated reduction of retinal BDNF level in diabetic VEGFR2 knockout mice [27] suggests that VEGF signaling in MCs might serve as a counter-balance for diabetes-downregulated production of neurotrophins, such as BDNF, in protecting retinal neurons in DR and hypoxia. We thus hypothesized that VEGFR2-mediated VEGF signaling in MCs was a master regulator for neuroprotection in DR and hypoxia, through its own downstream target, such as AKT survival signal, and the action of other trophic factors. To test this hypothesis and to determine whether VEGF signaling was the cause of an accelerated reduction of retinal BDNF in diabetic VEGFR2 knockout mice, we carried out this study. Our primary goal was to address whether VEGF played a direct role in mediating MC viability and neuroprotection through a classic neurotrophin BDNF, which is an important question in the field of neuroprotection in DR and hypoxic retinal diseases, such as AMD. In our hands, VEGF played a prominent role in promoting MC survival and proliferation, which led to an increase of MC-mediated BDNF production under diabetes-like conditions. These results, along with those from our previous work [27,36,46], indicate VEGF’s capability in upregulating BDNF production, presumably by making MCs healthier in a diabetic and/or hypoxic environment, which results in better neuroprotection by supporting more production of trophic factors, such as BDNF, as shown in our working hypothesis (Figure 4). Why is it important to reveal the role of BDNF in neuroprotection in DR and hypoxic retinal disorders? Although VEGF is a master regulator for neuroprotection in DR and hypoxic retinal diseases, the fact that VEGF is a major therapeutic target for these disorders made it impossible to act as a neuroprotective agent. Our finding suggests that BDNF, along with other MC-derived neurotrophic factors, are likely major candidates for the treatment of neuronal degenerations associated with DR and hypoxic retinal disorders [47]. Given that a substantial portion of patients with long-term anti-VEGF therapies appeared to have very thin retinas [16,19], a key characteristic of retinal degeneration, identifying neuroprotectant, such as BDNF which is a VEGF downstream target with no apparent harm to the retina, is paramount to the improvement of anti-VEGF therapies.

It has been proposed that MCs are a major cellular source of BDNF [48], which has been used in clinical trials for retinal neuroprotection in various non-DR-related retinal degenerations for decades [47,49,50] and is now being considered for clinical trial for DR [51]. Therefore, there is no question about the safety profile of BDNF, which exerts its trophic function through canonical neurotrophin receptor TRKs via PI3 kinase/AKT and ERK survival and proliferative pathways [52,53]. These signaling biomolecules have been shown to play an important role in mediating MC viability by nerve growth factor, a BDNF family neurotrophin [54]. In our hands, rBDNF was capable of stimulating rMC1 cell viability in a dose-dependent manner only under diabetic condition (Figure 3A). This result suggests that BDNF is a potential candidate for retinal MC viability and neuroprotection in clinical applications. To determine the potential mechanism of BDNF-mediated MC viability, we targeted TRK-B, the canonical BDNF receptor, with a siRNA knockdown approach, which resulted in the reduction of the activated AKT and ERK, the classical signatures for survival and proliferative pathways, in MC extracts. While there is no statistical significance in the reduction of total AKT in *TrkB* siRNA targeted MCs, we often observed some degree of total AKT reduction in our hands. This may represent the difference in disrupting the signaling between VEGF and BDNF. Finally, our finding that rBDNF was capable of rescuing the loss of *TrkB* siRNA transfected rMC1 cells under diabetic conditions (Figure 3F) suggests that the diabetes-accelerated reduction of BDNF is likely a contributing factor for the loss of MCs in MC-specific VEGFR2 knockout mice, eventually resulting in more neuronal degeneration [27]. The experiments described in Figure 3 are necessary to help us to reach such a conclusion. This observation also suggests a strong possibility of the existence of at least another mechanism for BDNF-mediated MC viability, in addition to BDNF/TRK-B signaling for MC maintenance in DR. Of note, the role of BDNF in promoting MC viability and neuroprotection is likely not equal to that of VEGF, as proposed recently [51]. This is why BDNF and VEGF may support MC viability in an addictive or synergistic fashion [36]. At present, we are also working on the potential role of glial cell line-derived neurotrophic factor (GDNF) in MC viability and neuroprotection [27]. This neurotrophin was downregulated in diabetic MC-specific VEGFR2 knockout mice, which may also play a synergistic role with VEGF or BDNF in supporting MC viability [9,36]. However, the underlying mechanisms of GDNF-mediated MC viability are most likely very different from that of BDNF [31]. The presence of these compensatory mechanisms provides an opportunity to find treatment strategies for neuronal degeneration in DR and other hypoxic retinal diseases in which VEGF is a therapeutic target for BRB breakdown.

In this study, we also provided evidence that VEGF is a potent agent to induce MC proliferation (Figure 1), which is relatively new to the field. At present, a major initiative in retinal regeneration is de novo neurogenesis from MCs [55], which definitely requires the proliferation of MCs. Since inducing MC proliferation with VEGF is much milder than what is used currently, our work could be used as a guideline for research in MC-derived neurogenesis. Finally, our experimental system can be easily adopted for rapid high-throughput drug tests for MC viability with simultaneous genetic analysis, as well as for other functional analysis in cell culture systems capable of efficient siRNA transfection.

## 5. Conclusions

In summary, we have made progress in the understanding of VEGF-mediated MC survival and its implication to neuroprotection in diabetes, demonstrated in our working hypothesis in Figure 4. Our results, along with those reported in our previous work [27,36,46], clearly identify that BDNF, is a VEGF downstream neuroprotectant that is capable of supporting MC and retinal neurons in diabetic and/or hypoxic environment. Since BDNF’s safety profile is undisputable, as demonstrated in the literature, it is a potential candidate for treating retinal neuronal degeneration in DR and hypoxic retinal diseases in which VEGF is a therapeutic target for BRB breakdown. As a substantial portion of long-term anti-VEGF drug-treated patients appeared to have retinal thinning, a pathological characteristic of retinal degeneration [16,19], BDNF and/or other neurotrophins may be particularly useful in improving the safety of long-term anti-VEGF treatment for BRB disorders.

## Figures and Tables

**Table 1:** Antibody dilution factors in immunoblotting (IB) and immunohistochemistry ((HC).

Target	Primary Antibody Dilution	Secondary Antibody Dilution
β-Actin (IB)	1:5000	1:4000
AKT (IB)	1:1500	1:4000
pAKT (IB)	1:2000	1:4000
BDNF (IB)	1:1000	1:4000
proBDNF (IB)	1:1000	1:4000
ERK (IB)	1:1500	1:4000
pERK (IB)	1:2000	1:4000
TRK-B (IB)	1:1000	1:4000
BrdU (IHC)	1:1000	1:700
GS (IHC)	1:1000	1:700

## Data Availability

All data presented in this work are included in the article.
